# The Politics of Covid-19 Vaccine Distribution and Recognition

**DOI:** 10.3389/phrs.2021.1604343

**Published:** 2021-10-29

**Authors:** Muhammad Adil Ashraf, Ameer Muhammad, Yasir Shafiq

**Affiliations:** VITAL Pakistan Trust, Karachi, Pakistan

**Keywords:** LMICs, COVAX, inequity, inequalities, politics

## Abstract

With COVAX touted as the only platform that is built on equity and fairness, there is growing discontent and concern that the platform is falling short of its goals as COVID ravages across multiple countries. There are two serious issues that we address here. Firstly, COVID distribution principles and mechanisms need to be rethought in terms of a shift from private to global interests with a focus on prioritizing deliveries. Secondly, with multiple vaccines present, it is vital that countries recognize all of them, once proven safe and effective, to prevent any form of vaccine apartheid and discrimination.

The COVID-19 pandemic in its indiscriminate ravaging of economic, health, and social systems across the world prompted calls for unity and equity to characterize the global response. The momentum for a coherent, unified, and ethical global response was partially down to the recognition that unless everyone was safe, no one was safe [1]. As the leading international mechanism and framework against COVID-19, the Access for COVID-19 Tools (ACT) Accelerator was touted as the arena which would ensure equitable, effective, and swift responses against the pandemic [2]. While the ACT Accelerator has had multiple successes, its most significant pillar, COVAX, has fallen short of its goals [3]. This will continue to be the case until at least two major issues are addressed. Firstly, COVID vaccine distribution needs to be aligned with global interests rather than private ones. Secondly, vaccine recognition must be unified and just in comparison to the present system of unfair recognition patterns.

## Distributive Justice

**FIGURE 1:**
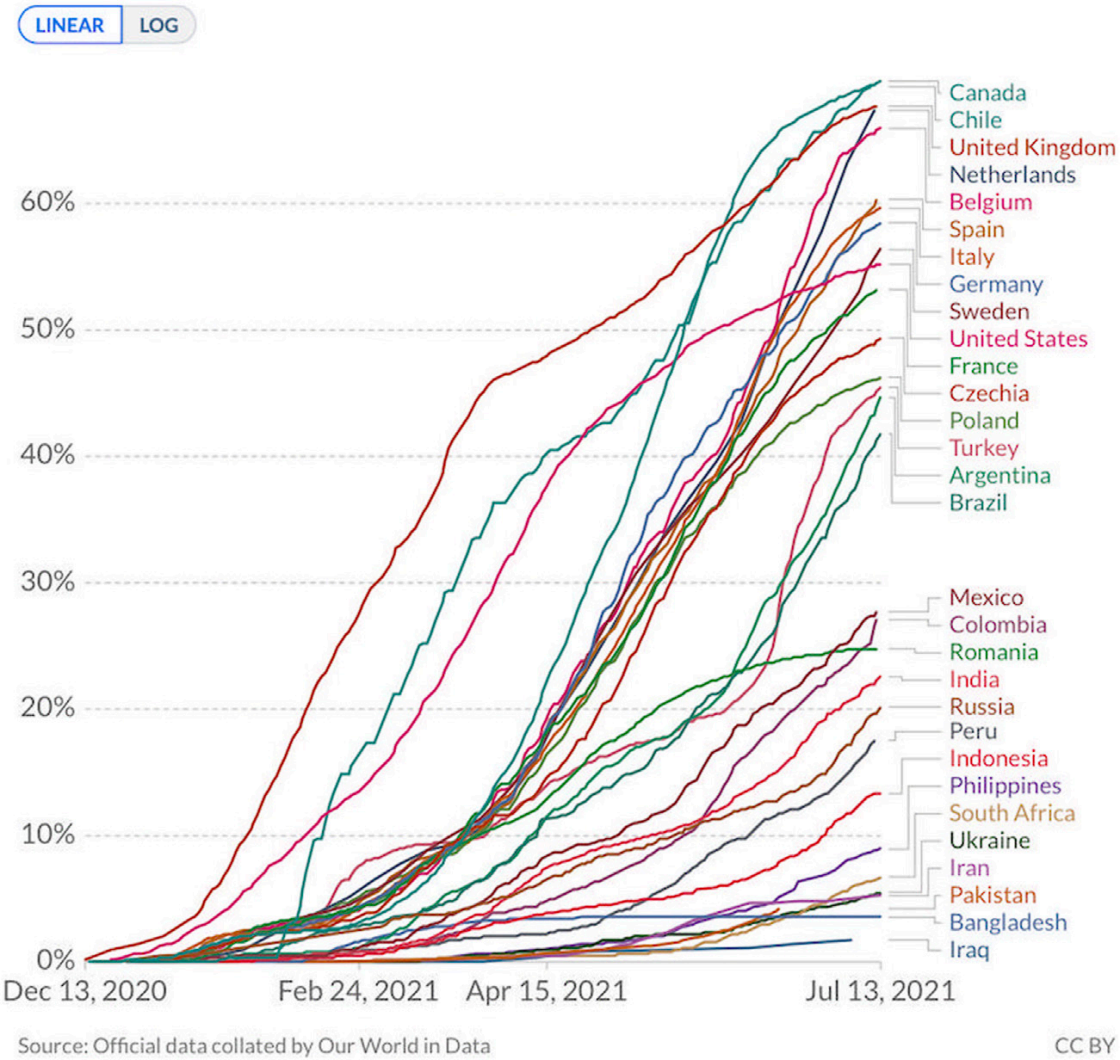
Countries comparison on ‘share of people vaccinated against COVID-19’ as of July 15, 2021 (Source: Official data collated by Our World in Data. Link: https://github.com/owid/covid-19-data/tree/master/public/data/vaccinations).

As of the second week of July 2021, around 24% of the world population had received at least one dose of the vaccine [4]. However, quite startingly, only 1% of people in developing countries were vaccinated. To put the immense divide in vaccinations into perspective, among the top 30 countries most COVID-19 affected countries, 69.6% of Canadian population has received at least once dose of vaccine versus 1.8% of the population in Iraq [4] (Figure 1). The numbers, on face value, reflect the structural inequalities and inequities in built within the global health order which is an extension of historical political and economic divides [5].

There are deep structural issues within and outside COVAX. By the end of March, COVAX was to distribute 100 million vaccines but it was only able to distribute 38 million [6]. Its promise of vaccinating 20% of all countries overlooks the fact that the pandemic reality is more complex than simply population sizes [2]. A distribution mechanism primarily based on population size is not prudent for it ignores the pressing emergencies of countries [7]. It would only be logical that COVAX develop a fluid and flexible prioritization mechanism rather than a fixed and rigid metric. There have already been cases of COVAX-provided vaccines being sent back and wasted in African countries, where the pandemic is still relatively mild, while cases were skyrocketing across other countries [8].

While these issues within COVAX render it far from perfect, it is vital that for a concentrated and unified vaccine delivery mechanism, COVAX be the priority rather than bilateral deals. The reality, however, is that countries have largely overlooked COVAX in their donation drives. Rather than providing vaccines to COVAX, a global institution especially responsible for the task, multiple countries have prioritized bilateral deals. Doing so allows them to secure important private geostrategic and economic gains in the name of vaccine diplomacy [9]. This explains why COVAX has only been able to distribute only 4% of the 1.95 billion doses that have been used worldwide [3]. Countries like China and India have donated their domestic vaccines in line with their own foreign policy objectives [7]. What all of this does is that it sidelines COVAX, pushes equity, fairness, and need pressures aside and prioritizes private, bilateral interests over global efforts to get rid of the pandemic. Just because COVAX is not perfect does not entail that it be overlooked in favor of bilateral deals. Rather, the focus should be on remedying its issues as outlined above.

### Recognizing Vaccines

Following from the negative role being played by countries in preventing an effective COVAX-led vaccination distribution system, the role of pharmaceutical companies must also be understood. As private entities, prioritizing profit, pharmaceutical companies understand the immense value of their patented products. To that end, they find it much more financially beneficial to strike bilateral deals with individual countries. This is why Pfizer decided only to sell 2% of its vaccines to COVAX and they will only arrive in the second half of the year [3]. Similarly, Moderna, despite promising 500 million doses, will only provide vaccines after October with the majority being given next year. Thus, COVAX has been largely dependent on the AstraZeneca vaccine which constitutes 95% of all its deliveries.

The AstraZeneca vaccine is being manufactured in the United Kingdom and at the Serum Institute of India. The Indian-manufactured, known as Covishield, is similar to the one made in Europe and is the majority vaccine being delivered to developing world under COVAX [10]. Recognizing vaccines is a deeply significant task. It has deep repercussions for traveling, economic linkages, vaccine hesitancy, and foreign policy gains. While the EU has recognized those vaccines which have been administered largely in the developed world, it initially refused to do so with Covishield even though the vaccine is similar to the one manufactured in Europe. Similarly, while some countries recognize Russian, and Chinese vaccines, they are currently not recognized by a host of Western countries, including EU ones and the US, which raises questions regarding unfairness in travel restrictions. Saudi Arabia, which hosts millions of migrant laborers, also requires a booster shots of the leading Western vaccines even if an individual is fully vaccinated by Sinovac or Sinopharm. Accessing these Western vaccines is quite difficult for individuals in developing countries where supply is limited.

There are fears that a discriminate system of vaccine recognition will lead to further vaccine hesitancy. Studies have already shown that the developing world, specially African countries, have quite high vaccine hesitancy rate [8]. Furthermore, through a discriminate recognition system, all efforts of a unified, coherent, and fair vaccine procurement and distribution system under COVAX will be discredited. How COVAX will maintain and uphold its legitimacy if most of its vaccines are not recognized by so many countries? Such disputes will only add fire to an already fragile multilateral global effort. Lastly, there is no reason or justification for countries to employ selective recognition biases when the WHO and multiple regulatory bodies have passed the Chinese and Covishield vaccines for their efficacy levels and ability to save significant number of lives. What is explicitly needed is a need for all and only WHO approved vaccines to be unanimously recognized by all countries to allow for a uniform, coherent, and consistent vaccine recognition system.

## Abbreviations

ACT-19, Access for COVID-19 tools accelerator; Covid-19, Coronavirus disease 2019
